# Monitoring Malaria: Genomic Activity of the Parasite in Human Blood Cells

**DOI:** 10.1371/journal.pbio.0000011

**Published:** 2003-08-18

Every year, malaria kills as many as 2.5 million people. Of these deaths, 90% occur in sub-Saharan Africa, and most are children. While four species of the single-celled organism Plasmodium cause malaria, Plasmodium falciparum is the deadliest. Harbored in mosquito saliva, the parasite infects its human host as the mosquito feeds on the victim's blood.

Efforts to control the disease have taken on an increased sense of urgency, as more P. falciparum strains show resistance to antimalarial drugs. To develop new drugs and vaccines that disable the parasite, researchers need a better understanding of the regulatory mechanisms that drive the malarial life cycle. Joseph DeRisi and colleagues now report significant progress toward this goal by providing the first comprehensive molecular analysis of a key phase of the parasite's life cycle.

While P. falciparum is a single-celled eukaryotic (nucleated) organism, it leads a fairly complicated life, assuming one form in the mosquito, another when it invades the human liver, and still another in human red blood cells (erythrocytes). The intraerythrocytic developmental cycle (IDC) is the stage of the P. falciparum lifecycle associated with the clinical symptoms of malaria. Using data from the recently sequenced P. falciparum genome, the researchers have tracked the expression of all of the parasite's genes during the IDC.

The pattern of gene expression (which can be thought of as the internal operating system of the cell) during the IDC is strikingly simple. Its continuous and clock-like progression of gene activation is reminiscent of much simple life forms—such as a virus or phage—while unprecedented for a free living organism. Virus and phage behave like a “just-in-time” assembly line: components are made only as needed, and only in the amount that is needed. In this respect, malaria resembles a glorified virus.

Given the remarkable coupling of the timing of gene activation with gene function, as shown in this paper, this understanding could help identify the biological function of the 60% of genes in P. falciparum that encode proteins of unknown function.

P. falciparum appears to be ultra-streamlined and exquisitely tuned to perform a single job: consume, replicate, and invade. The simple program regulating the life of P. falciparum may hold the key to its downfall as any perturbation of the regulatory program will likely have dire consequences for the parasite. This offers renewed hope for the design of inhibitory drugs targeted at the regulatory machinery that would irreparably foul the parasite's regulatory program, ultimately resulting in its death.

**Figure:**
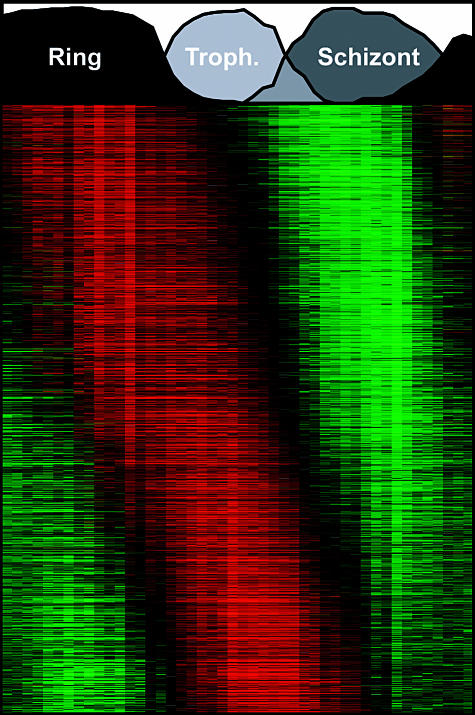
Gene expression profile of P. falciparum

